# Portable and sensitive air pollution monitoring

**DOI:** 10.1038/s41377-018-0017-x

**Published:** 2018-05-18

**Authors:** Judith Su

**Affiliations:** Department of Biomedical Engineering and College of Optical Sciences, University of Arizona, Tucson, AZ 85721 USA

**A compact optical nanofiber sensor enables detection of 100 nm particles in the field. This device improves our ability to track air quality with high spatiotemporal resolution.**

**Fig. 1:**
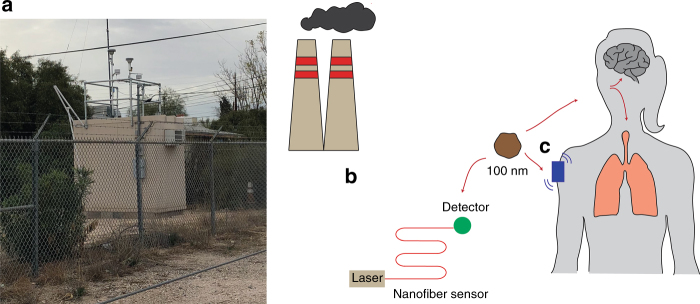
Air pollution monitoring. **a** Photograph of an air monitoring station in Tucson, Arizona. These stations draw in air and size separate particulate matter using an impactor. PM_2.5_ particles are collected on a filter. The filter is weighed to determine the mass of PM_2.5_ particles collected for a given volume of sampled air. Due to large spatiotemporal variations in air pollution, portable sensors which can sense someone’s personal environment throughout the day are desired. Ultrafine particles can go directly into the lungs and brain causing cancer and Alzheimer’s. **b** A compact serpentine nanofiber sensor can detect 100 nm particles over the period of 1 month. **c** A future wearable air pollution monitoring system could be part of an armband or smart watch that tracks personal exposure levels.

Air pollution has enormous health and economic costs, causing an estimated 7 million deaths per year^1^ and costing US$225 billion globally in 2013 according to the most recent data from the World Health Organization (WHO) and World Bank, respectively^2^. Despite this impact, air pollution levels continue to increase, with 92% of the world’s population living in environments in which WHO air quality guidelines are not met^3^. In the United States, the Environmental Protection Agency (EPA) regulates six criteria air pollutants, one of which is particulate matter (PM)^4^. While portable sensors for particles under 2.5 μm in diameter (known as PM_2.5_) exist, compact sensor development for ultrafine particles, which are <100 nm in diameter, has lagged. Ultrafine particles are unregulated by the EPA, and due to their small size, they can directly enter the lungs or the brain and have been linked to cancer and Alzheimer’s, among other diseases^5^. The main source of ultrafine particles is exhaust from motor vehicles, combustion, and industrial processes^6^. Despite local variations in air pollution levels^7^ for both fine and ultrafine particles, air monitoring systems are few and far between, numbering approximately 2–3 stations for cities of 1,000,000 people or more in the United States^8^. Furthermore, such monitoring systems are large (Fig. 1a), cumbersome to install, and expensive, costing from US$6,000 to US$36,000^9^, and they can be affected by highly localized factors, such as nearby trees, making their measurements potentially unrepresentative of the air quality even a few blocks away. There is a need for personal, low-cost, portable, and sensitive systems that are capable of long-term, continuous, routine, and real-world environmental monitoring.

Despite the need for personal sensors, there is often a size and sensitivity trade-off^9^. Large, stationary stations can provide more accurate detection data than mobile portable systems. Yun-Feng Xiao’s group from Peking University has created a compact and sensitive serpentine nano-optical fiber sensor (Fig. 1b) that can operate in an open environment^10^. This sensor is capable of counting and sizing particles smaller than 100 nm; this diameter represents the threshold of the ultrafine particle designation. In addition, Xiao’s sensor has a sizing resolution of 10 nm, indicating that the approach could probably be used to sense particles smaller than 100 nm. Due to the amount of light from the nanofiber that can interact with a particle, the upper particle size limit that their sensor can detect is approximately 1 µm. This detection limit provides coverage of the full range of particles involved in the PM_1.0_ count; the group has shown that this count correlates with the concentration of PM_2.5_ particles in empirical experiments.

The serpentine nano-optical fiber sensor measures particulate concentrations by monitoring step-wise drops in the optical power transmitted through the fiber as particles land. These changes in power can be related to the size of the particle that has landed on the fiber using Rayleigh–Gans scattering theory^11^. The serpentine configuration of the nanofiber helps to increase the capture area of the sensor. In addition to detecting and counting the number of particles within a given size range designation (PM_2.5_, PM_1.0_, etc.), Xiao’s sensor can also provide highly precise size information, which can help to better establish correlations between pollution composition and health hazards and can inform future environmental regulations.

The sensor was used for particulate matter monitoring in Beijing over a period of a month, and the data agree well with official data from the Beijing Municipal Environmental Monitoring Center, demonstrating that the sensor can be used for real-world, long-term monitoring. In these experiments, measurements were taken every few hours. Unlike other precise particle sensing systems^12^, Xiao’s sensor does not require a tunable laser, which makes this system cheaper and more portable.

The future of air pollution monitoring technology is low cost, chemically specific, sensitive, weather protected, mobile sensor arrays capable of being worn or attached to existing infrastructure (Fig. 1c). When used together, such sensors could form an early warning network through a “crowdsourced” map of outdoor pollutant levels. These sensors would have low power consumption and would be able to provide continuous, long-term, live monitoring of pollutant measurements in dynamic and harsh environments. Additional considerations, such as data storage, management, dissemination, and privacy, would also need to be addressed. These sensors could provide an abundance of hyper local air quality information, such as data related to household chemical use or air pollutants in car passenger cabins. The data obtained could be linked to indicators of health and be used to quickly inform the public as to their risk for illnesses, such as asthma, bronchitis, cancer, and Alzheimer’s.
